# Reviewers in 2018

**DOI:** 10.1098/rsob.190032

**Published:** 2019-03-06

**Authors:** David Glover

**Affiliations:** Editor-in-Chief

The Editors of *Open Biology* would like to thank all reviewers who contributed their time by refereeing manuscripts submitted throughout 2018. From previous feedback, we know that most reviewers value recognition of their efforts by the journal and through services, such as *Publons*, which is integrated with *Open Biology*.

To provide an opportunity for recognition, we list the names of all reviewers who opted in. This article is made permanent and citeable by its digital object identifier (DOI). We encourage you to quote your contribution in your applications for tenure and grants or other forms of research assessment. Where you have provided it, we have included your ORCID, so your contribution can be unambiguously assigned to you.

The team really do appreciate all the work our reviewers do on behalf of the journal, and we look forward to working with you again in 2019.

Harwood A

Lefebvre P

Royle S http://orcid.org/0000-0001-8927-6967

Ceravolo G

Fejtova A

Faeder J

Antoniazzi M

Golemis E

Masu M

Gutleb A

Luders J

Torriglia A

Sanchez-Mazas A

Primig M

Pieters J

Edlich F

Du L-L http://orcid.org/0000-0002-1028-7397

West S

Parlato R

Beck S

Zikova A

Basu U

Gilmore A http://orcid.org/0000-0002-9988-0436

Flaus A

Cavodeassi F

Gough J

Jang S-W

Kelsey KT

Fernández HLÁ

Boto L

Zachos N

Goedert M

Hartsuiker E

Spaapen R

Pancione M

Root D

Popławski T

Church D

Okada M

Carr G

Miao Y-G http://orcid.org/0000-0002-0545-7312

Merdes A http://orcid.org/0000-0002-3739-2728

Hullar M

Ehrenhofer-Murray AE

Anton PM

Gergely F

Stanley-Wall N

Maeshima K

Mizuno K

Schafer W http://orcid.org/0000-0002-6676-8034

López-Camarillo C

Nikonova A

Hastings K

Macia A

Lafanechère L

Giangrande A

Winey M

Adelson D

Wray S

Ischebeck T

De Summa S

Bacanli M

Sardanyes J

Bártek J

Hogg P http://orcid.org/0000-0001-6486-2863

Ahnert S http://orcid.org/0000-0003-2613-0041

Benaroch P

Tomida J

Valenzuela R

Mathew A

Rad R

Chen T

Lishko P

Liu F

Schmidt C http://orcid.org/0000-0003-4627-8326

Schmid M

McKenney R

Whitworth A

Nilsson J

Varma D

Gaestel M

Hinman J

Poliseno L

Prigent C

Gu Y

Hunter T

Ribas L

Hardwick K

Koch K-W

Blom B

Oft M

Harris J

Gao G

Ewer J

McKean P

Mamedova L

Juhasz G

Tomokiyo A

Chang B http://orcid.org/0000-0002-6525-4429

Robinson D

Liu W

Dodson C

Jiang J

Ryter SW

Feng W-N

Daugaard M

Johnson P

Yang X

Tissir F

Gallouzi I

Kaushansky A

Pramfalk C

Liwen J

Fernandez-Gonzalez R

Katz P http://orcid.org/0000-0003-0786-1287

Riabinina O

Marchio C

Paterson A

Hindmarch C

Shelkovnikova T

Li L

Hendriks R

Ceruti S

Garret M

Johnston L

Woolley J

Blum D

Kurusu T

Zhou X http://orcid.org/0000-0002-2385-8224

Szczesna-Cordary D

Larschan E

Bresnick E

Rivera-Pomar R

Lau N

Warr C

Breitling R

Smibert C

Graham A

Waskiewicz A

Famulski J

Loo Y

Barbash D

Gimelbrant A

Mancuso M

Potter C http://orcid.org/0000-0002-5223-8112

Chaveroux C

Lomakin A http://orcid.org/0000-0002-5346-3426

Faissner A

Robertson H

Chen W

Dai Z

Xu J

Mostowska A http://orcid.org/0000-0003-4109-5125

Ding W

Liew WC http://orcid.org/0000-0001-9354-1647

Ridgway G http://orcid.org/0000-0002-7676-7860

Wilson I

Yamamoto K

Shayakhmetov D

Llimargas M

Mungall C

Puthiyaveetil S

